# Whole Transcriptome Analysis Provides Insights into Molecular Mechanisms for Molting in *Litopenaeus vannamei*

**DOI:** 10.1371/journal.pone.0144350

**Published:** 2015-12-09

**Authors:** Yi Gao, Xiaojun Zhang, Jiankai Wei, Xiaoqing Sun, Jianbo Yuan, Fuhua Li, Jianhai Xiang

**Affiliations:** 1 Key Laboratory of Experimental Marine Biology, Institute of Oceanology, Chinese Academy of Sciences, Qingdao, China; 2 University of Chinese Academy of Sciences, Beijing, China; Chang Gung University, TAIWAN

## Abstract

Molting is one of the most important biological processes in shrimp growth and development. All shrimp undergo cyclic molting periodically to shed and replace their exoskeletons. This process is essential for growth, metamorphosis, and reproduction in shrimp. However, the molecular mechanisms underlying shrimp molting remain poorly understood. In this study, we investigated global expression changes in the transcriptomes of the Pacific white shrimp, *Litopenaeus vannamei*, the most commonly cultured shrimp species worldwide. The transcriptome of whole *L*. *vannamei* was investigated by RNA-sequencing (RNA-seq) throughout the molting cycle, including the inter-molt (C), pre-molt (D0, D1, D2, D3, D4), and post-molt (P1 and P2) stages, and 93,756 unigenes were identified. Among these genes, we identified 5,117 genes differentially expressed (log_2_ratio ≥1 and FDR ≤0.001) in adjacent molt stages. The results were compared against the National Center for Biotechnology Information (NCBI) non-redundant protein/nucleotide sequence database, Swiss-Prot, PFAM database, the Gene Ontology database, and the Kyoto Encyclopedia of Genes and Genomes database in order to annotate gene descriptions, associate them with gene ontology terms, and assign them to pathways. The expression patterns for genes involved in several molecular events critical for molting, such as hormone regulation, triggering events, implementation phases, skelemin, immune responses were characterized and considered as mechanisms underlying molting in *L*. *vannamei*. Comparisons with transcriptomic analyses in other arthropods were also performed. The characterization of major transcriptional changes in genes involved in the molting cycle provides candidates for future investigation of the molecular mechanisms. The data generated in this study will serve as an important transcriptomic resource for the shrimp research community to facilitate gene and genome annotation and to characterize key molecular processes underlying shrimp development.

## Introduction

The exoskeleton of Crustacean is essential for body shape maintenance, defense response, and locomotion via attached somatic muscles. Nevertheless, this structure can confine body growth and restrict mating. Thus, Crustacean periodically shed and replace their old exoskeletons, a process referred to as molting [1]. Shrimp is an important kind of Crustacean, and molting is a crucial process for shrimp. Molting, or ecdysis, allows the shrimp body to expand rapidly by absorbing water after every molt, but produces little or no increase in body size (volume) until the next ecdysis. Thus, shrimp grows in a ladder-type manner [2]. In theory, more frequent molting would result in larger body size. In addition, molting is a vital step in metamorphosis. During the early stages of their life cycle, peneaid shrimp undergo metamorphosis in four larval stages: nauplii, zoea, mysis, and post-larvae. Molting occurs twelve times more frequently during these larval stages than in later stages of the shrimp life cycle [3]. Reproductive molting is also necessary for shrimp to mate and spawn. On an average, penaeid shrimp, such as *Litopenaeus vannamei*, experience about 50 molts during a lifetime [4]. Molting not only shapes shrimp morphology, physiology, and behavior, but also plays roles in deformities, death, and predation. Moreover, molting can slough off attachments and parasites, and also affect limb regeneration [5]. Therefore, it is very important to understand the mechanisms underlying shrimp molting.

Molting activity persists throughout the life of a shrimp, and has clear physiological effects. The molting cycle can be divided into four recurrent stages, including inter-molt, pre-molt, ecdysis, and post-molt, according to the appearance of epidermis, pigmentation, formation of new setae, and the presence of matrix or internal cones in the setal lumen, respectively [5,6]. During the inter-molt period (C), the muscle regenerates and energy is stored as glycogen and lipids [7]. The pre-molt phase (D), generally subdivided into D0, D1, D2, D3, and D4, is the longest period and comprises up to two-thirds of the molting cycle. Shrimp lose the connection between living tissue and extracellular cuticles at early pre-molt. Subsequently, somatic muscle atrophy, resorption of the old exoskeleton, and formation of a new exoskeleton under molting fluid then occur in preparation for ecdysis [8,9]. Ecdysis takes only a few minutes and individual is able to escape from the confines of the cuticle and take up water so the body expands quickly expand. After ecdysis (ecdysis-P1 and P1-P2), there is a rapid increase in body size, followed by a period with little or no body size increase until the next molt [10]. The newly formed exoskeleton expands through mineral and protein deposition and quickly becomes hard for defense and locomotion during post-molt.

Molting is a complex process under the control of many regulatory factors, including neuropeptide hormones, ecdysteroids [11–13], and the external environment [14–16]. Previous studies on shrimp molting were mainly based on eyestalk ablation, hormone isolation, and cloning, while the expression analyses of genes associated with exoskeleton hardening involved quantitative reverse transcription polymerase chain reaction (RT-PCR) and microarrays [17–20]. For instance, three cDNA fragments from genes associated with synthesis of molting-inhibiting hormone (MIH) were isolated from eyestalk in three crustaceans: *Eriocheir sinensis*, *Trachypenaeus curvirostris*, and *Fenneropenaeus chinensis* [21–23]. Two retinoid X receptor (RXR) genes, an ecdysone receptor gene (*ECR*), and an ecdysone inducible gene (*E75*), were identified in *F*. *chinensis* [17,18]. Distinctive MIH-like peptides, which have been implicated in repression of ecdysteroid synthesis, were identified in Kuruma shrimp *Marsupenaeus japonicus* [24,25]. Recently, studies investigating expression patterns of *LvEcR*, *LvRXR*, and *LvE75* in different tissues and developmental stages in *L*. *vannamei* have found that molting signaling in different tissues exhibits different expression patterns, which appear to be reflective of their distinct functions in molting, chitin metabolism, and muscle growth [26]. Despite these previous findings, our understanding of the molecular mechanisms underlying shrimp molting remains very limited.

In order to connect the mechanisms and molecular events associated with shrimp molting, we used RNA-Sequencing (RNA-seq) to investigate expression changes across all genes during the molting process of *L*. *vannamei*, one of the most commonly cultured shrimp species, representing nearly 40% of penaeid shrimp production worldwide [27]. This approach is considered to have high power to dissect gene networks associated with particular biological and developmental processes at a whole transcriptome level [28–30]. Here, we report a comprehensive analysis of global transcript expression changes associated with shrimp molting, which not only enhances our understanding of the molecular mechanisms underlying shrimp molting, but also provides a valuable resource of transcriptome data shrimp genome annotation and further identification of candidate genes controlling molting in shrimp and other molting animals.

## Materials and Methods

### Sample collection and RNA isolation

Healthy adult Pacific white shrimp *(L*. *vannamei*) with an average body length of 14–16 cm were collected from laboratory culture ponds. All shrimp were the same generation and had been cultured over six months to minimize generational and environmental effects. All procedures involving animals throughout the experiments were conducted in strict accordance with the Chinese Legislation on the Use and Care of Laboratory Animals. And all animal experiments were performed as per the institutional ethic committee guidelines of Institute of Oceanology Chinese Academy of Sciences (IOCAS).

**Fig 1:**
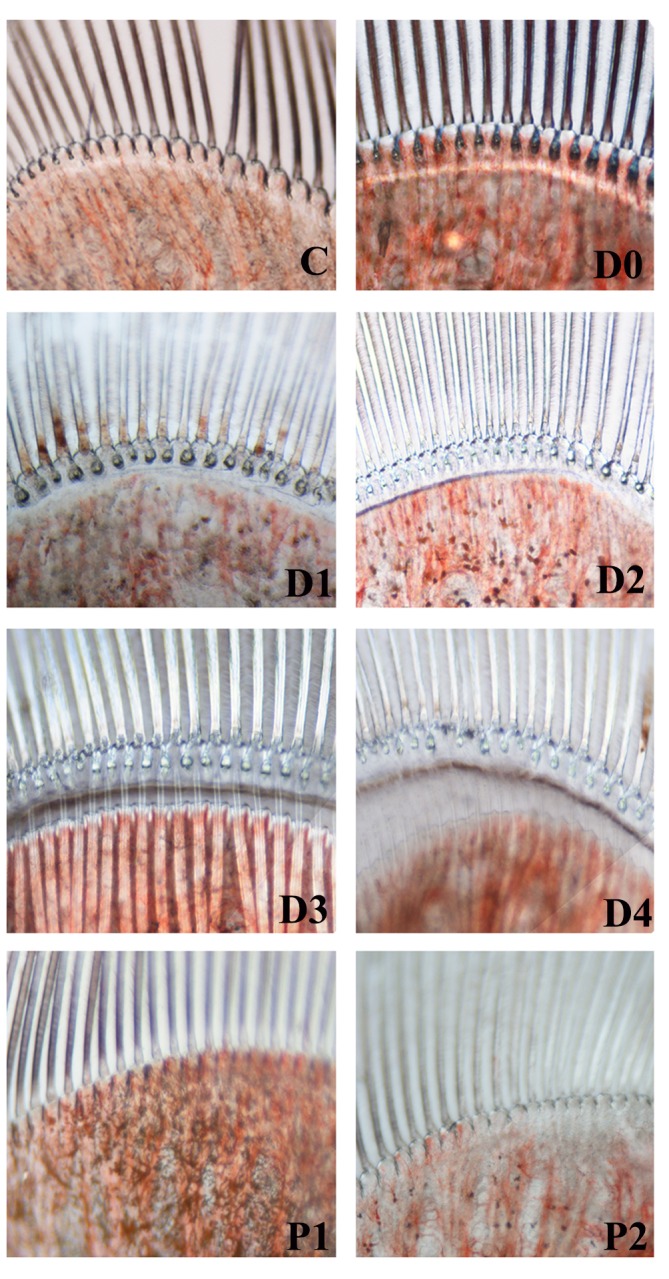
Morphological changes of shrimp uropods during molting stages under a light microscope (400 × magnification). Observed physical characteristics of each stage were as follows: 1) Inter-molt stage (C stage): mature setal cones and fully-spread epidermis. 2) Pre-molt stage (D stage): D0, a clear margin of epidermal tissue at the base of the setal cones. D1, a clear, narrow zone between the setal cones and the epidermis. D2, a wider clear zone and a wavy edge of epidermis. D3, a wider clear zone, highly wavy edge of epidermis, and a thin, white layer at the edge of the epidermis. D4, a noticeably wider clear zone, serrated edges of epidermis, a light-reflecting white layer at the edge of the epidermis, and parallel-band edepidermis. 3) Post-molt stage (P stage): P1, soft and delicate setae, absence of setal cones. P2, presence of young setal cones.

Animals were sorted by molt stage according to the appearance of epidermis, pigmentation, formation of new setae, and presence of matrix or internal cones in the setal lumen (Fig 1). Molting stages were classified as inter-molt (C), pre-molt (D0, D1, D2, D3, and D4), and post-molt (P1 and P2). A loop design comparing consecutive molt stages (C-D0-D1-D2-D3-D4-P1-P2-C) was employed for the analysis to achieve consecutive times for all periods without interruption. Each whole shrimp was considered a separate sample and two samples were taken as biological replicates for each molt stage. All samples were immediately frozen in liquid nitrogen and stored at -80°C until RNA isolation.

Each sample was crushed in a mortar with liquid nitrogen, and total RNA was prepared using a Trans-up RNA isolation kit (Biostar, Shanghai, China) according to the manufacturer’s protocol. The yield and purity of each RNA sample were determined using a NanoDrop^™^ 2000 spectrophotometer (Thermo Scientific, USA), and the integrity of all RNA samples was assessed by gel electrophoresis with 1% agarose. Total RNA was treated with DNase to remove DNA contamination.

### RNA sequencing library construction and Illumina sequencing

Isolation and enrichment of mRNA from total RNA was performed using oligo (dT) magnetic beads (Illumina, CA, USA). Then, mRNA was fragmented to short fragments to be used as templates for random hexamer-primed synthesis of first strand cDNA by fragmentation buffer. Second-strand cDNA was synthesized using buffer, dNTPs, RNase H, and DNA polymerase I. A paired-end cDNA library was synthesized using the Genomic Sample Preparation Kit (Illumina, CA, USA) according to the manufacturer’s instructions. Short fragments were purified with QIAQuick^®^PCR extraction kit (QIAGEN, Germany) and eluted in 10 μL of EB buffer (QIAGEN, Germany). These short fragments were connected via sequencing adapters (Illumina, CA, USA). Agarose gel electrophoresis was used to select fragments approximately 50 bp in size. Finally, cDNA libraries were sequenced on an Illumina HiSeq^™^ 2500 (Novogene, Beijing).

### Assembly of sequencing data and gene annotation

Raw sequence data were transformed by base calling into sequence data and stored in fastq format. Raw reads were cleaned by removing adapter sequences, empty reads, and low quality sequences. Cleaned reads were assembled with the software package RSEM [31] using *L*. *vannamei* reference transcriptome data obtained from the molting-transcriptome sequencing (SRX1411196) and combined with the data previously sequenced by our laboratory (SRR1460493, SRR1460494, SRR1460495, SRR1460504 and SRR1460505).

For annotation analysis, unigenes were BLASTX-searched against five databases, including the National Center for Biotechnology Information (NCBI) non-redundant protein sequence (NR) database, the NCBI non-redundant nucleotide sequence (NT) database, KEGG Orthology (KO) database, Swissprot, and the PFAM database, using a cut-off E-value of 10^−5^. Unigenes were annotated based on BLASTX results, and the best alignments were used for downstream analyses.

### Normalized expression levels of genes from RNA‑seq

To eliminate the influence of different gene lengths and sequence discrepancies on expression calculations, gene expression levels based on read counts obtained by RSEM were normalized using the FPKM (Fragments Per Kilo bases per Million fragments) transformation [32]. Thus, calculated gene expression levels could be used for direct comparison among samples. Expression values were standardized across the dataset to enable the data from different genes to be combined.

### Screening of differential expression genes (DEGs)

Using the R package DEGseq, differentially expression genes (DEGs) were identified with a random sampling model based on the read count for each gene at different developmental stages [33]. False discovery rate (FDR) ≤0.001 and absolute value of log_2_ratio ≥1 were set as the threshold for significance of gene expression differences between adjacent samples (C-D0, D0-D1, D1-D2, D2-D3, D3-D4, D4-P1, P1-P2, and P2-C).

### Gene Ontology and KEGG analysis

Gene Ontology (GO) terms were used to describe biological processes, molecular functions, and cellular components. As an international standardized gene functional classification system, GO offers both a dynamically updated controlled vocabulary and strictly defined concepts to describe the properties of genes and their products comprehensively. The Blast2GO(version 3.0) (https://www.blast2go.com/) program was used to obtain GO annotations for all genes with a Fisher’s Exact Test (filtered with FDR ≤0.01) [34]. The unigene sequences were aligned to the Clusters of Orthologous Group (COG) database. Using GO functional classification analysis (WEGO), we categorized all genes based on function [35]. The Kyoto Encyclopedia of Genes and Genomes (KEGG) database was used to assign and predict putative functions and pathways associated with the assembled sequences [36]. A heat map which grouped genes according to FPKM values was generated in Cluster 3.0 [37] and visualized in TreeView 1.6 to analyze expression levels across molting periods [38].

### Real time qPCR amplification

To validate RNA-seq data and expression profiles, six genes were randomly selected for validation using real-time quantitative polymerase chain reaction (RT-qPCR). Actin T2 (c82047_g1) was used as an internal standard, and relative gene expression levels were calculated using the comparative Ct method with the formula 2^-ΔΔCt^ [39]. All samples were run in triplicate in separate tubes; each cDNA sample was run in duplicate. All data were expressed as mean +SD after normalization. Real-time qPCR results were then compared with transcriptome data to detect the expression correlation of each gene. The primers used for amplification and the annotations of the products are listed in S1 Table.

### Data Availability

The sequence data in this study have been deposited into the NCBI Sequence Read Archive (http://trace.ncbi.nlm.nih.gov/Traces/sra/sra.cgi?view=studies), and the accession numbers of the eight SRA samples are as follows: SRX1098368, SRX1098369, SRX1098370, SRX1098371, SRX1098372, SRX1098373, SRX1098374, and SRX1098375.

## Results

### Result of RNA-Sequencing and analysis

Sixteen normalized cDNA libraries prepared from all molting stages were sequenced using the Illumina Hiseq 2500 platform. After removal of adaptor sequences, duplicate sequences, ambiguous reads, and low-quality reads, a total of 148.25 millions of clean reads (7.41Gb) with 98.7% Q20 bases were selected as high-quality reads for expression analysis. An overview of the sequencing results is summarized in Table 1. Among the total number of reads from sixteen samples, 83.11% to 88.38% were matched in comparison with the reference genome. The percentages of mapped reads were similar in these libraries.

**Table 1:** An overview of sequencing and assembly of the shrimp transcriptome from *L*. *vannamei*.

Sample	Raw Reads	Clean reads	Total mapped	Clean bases	Error(%)	Q20(%)	GC(%)
**lvC_1**	8462163	8378139	7205223(86.00%)	0.42G	0.01	98.64	50.04
**lvC_2**	8356671	8281105	7228674(87.29%)	0.41G	0.01	98.68	50.01
**lvD0_1**	9729632	9658189	8372908(86.69%)	0.48G	0.01	98.63	50.14
**lvD0_2**	9001963	8949633	7698406(86.02%)	0.45G	0.01	98.74	49.85
**lvD1_1**	7069133	7010089	5949698(84.87%)	0.35G	0.01	98.52	46.72
**lvD1_2**	8289817	8237292	9226254(86.42%)	0.41G	0.01	98.58	46.87
**lvD2_1**	10758027	10676498	9226254(86.42%)	0.53G	0.01	98.66	49.69
**lvD2_2**	9878904	9801263	8596987(87.71%)	0.49G	0.01	98.74	48.46
**lvD3_1**	8732484	8665896	7228584(83.41%)	0.43G	0.01	98.65	51.18
**lvD3_2**	8414766	8350596	6939910(83.11%)	0.42G	0.01	98.68	50.47
**lvD4_1**	10154005	10078307	8667790(86.00%)	0.5G	0.01	98.79	50.70
**lvD4_2**	9650580	9598685	8199145(85.42%)	0.48G	0.01	98.81	50.08
**lvP1_1**	10570861	10500814	9255363(88.14%)	0.53G	0.01	98.71	50.09
**lvP1_2**	10017393	9937550	8650085(87.04%)	0.5G	0.01	98.69	50.77
**lvP2_1**	10205898	10135988	8787490(86.70%)	0.51G	0.01	98.78	49.61
**lvP2_2**	10073944	9993119	8832160(88.38%)	0.5G	0.01	98.70	49.97

By comparing with the reference transcriptome data, all clean reads were assembled into 93,756 unigenes. To understand potential genetic mechanisms undergirding *L*. *vannamei* molting, 93,756 assembled unigenes were BLASTX-searched against five databases. Of the 93,756 unigenes, 15,582 match to known proteins in the NR database, and 3,689 match to putative homologues in the NT database. The KO database provided annotation for 6,493 unigenes, the Swissprot database confirmed matches for 12,873 unigenes, and 20,784 unigenes found putative homologues in the PFAM database.

### Expression analysis and evaluation of gene expression

Expression levels for each unigene during the eight stages of molting are shown in S2 Table. The number of expressed unigenes (FPKM≥0.3) in each stage were 55,639 (C), 56,655 (D0), 60,115 (D1), 59,636 (D2), 50,693 (D3), 57,689 (D4), 52,813 (P1), and 58,890 (P2), respectively. Among the unigenes expressed in these eight stages, 3,995 (C), 4,096 (D0), 5,994 (D1), 6,664 (D2), 4,187 (D3), 5,254 (D4), 4,360 (P1), and 5,888 (P2), respectively, were found to be expressed stage-specifically. In contrast, a large number (28,508) of unigenes were found to be expressed at all eight stages. Some genes exhibited low variance or low expression levels, and were thought either to be housekeeping genes or expressed infrequently during the molting cycle.

### Analysis of differentially expressed genes (DEGs) among molting stages

**Fig 2:**
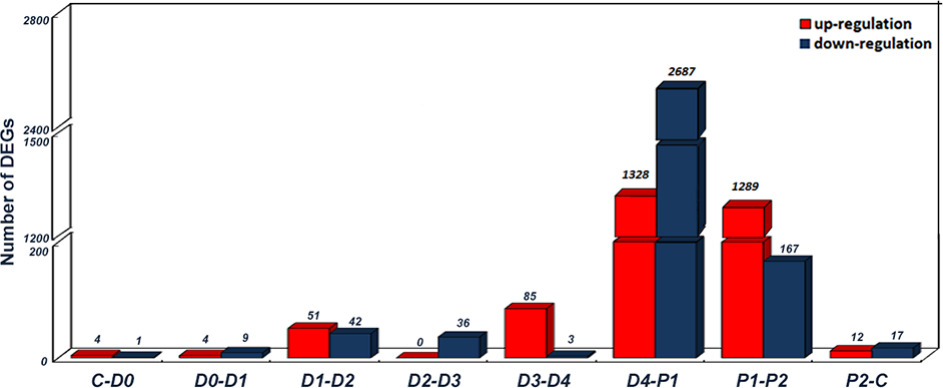
Differentially expressed genes (DEGs) detected between eight comparisons of adjacent molting stages (C-D0, D0-D1, D1-D2, D2-D3, D3-D4, D4-P1, P1-P2, and P2-C). The number of up-regulated and down-regulated genes between comparisons is given. The x-axis indicates adjacent stages in comparisons. The y-axis indicates the number of DEGs.

Our analysis targeted genes expressed at relatively high levels between adjacent molting stages. Using log_2_ratio ≥1 and FDR ≤0.001 as a threshold, a total of 5,117 differentially expressed genes (DEGs) between any two adjacent molting stages (i.e., C-D0, D0-D1, D1-D2, D2-D3, D3-D4, D4-P1, P1-P2, and P2-C) were identified (S3 Table). The numbers of up-regulated and down-regulated DEGs are shown in Fig 2. A total of 4,015 and 1,456 DEGs were detected between D4 and P1 and between P1 and P2, respectively, representing the two largest groups of DEGs among the eight comparisons.

### Hierarchical clustering of DEGs among the eight stages

**Fig 3:**
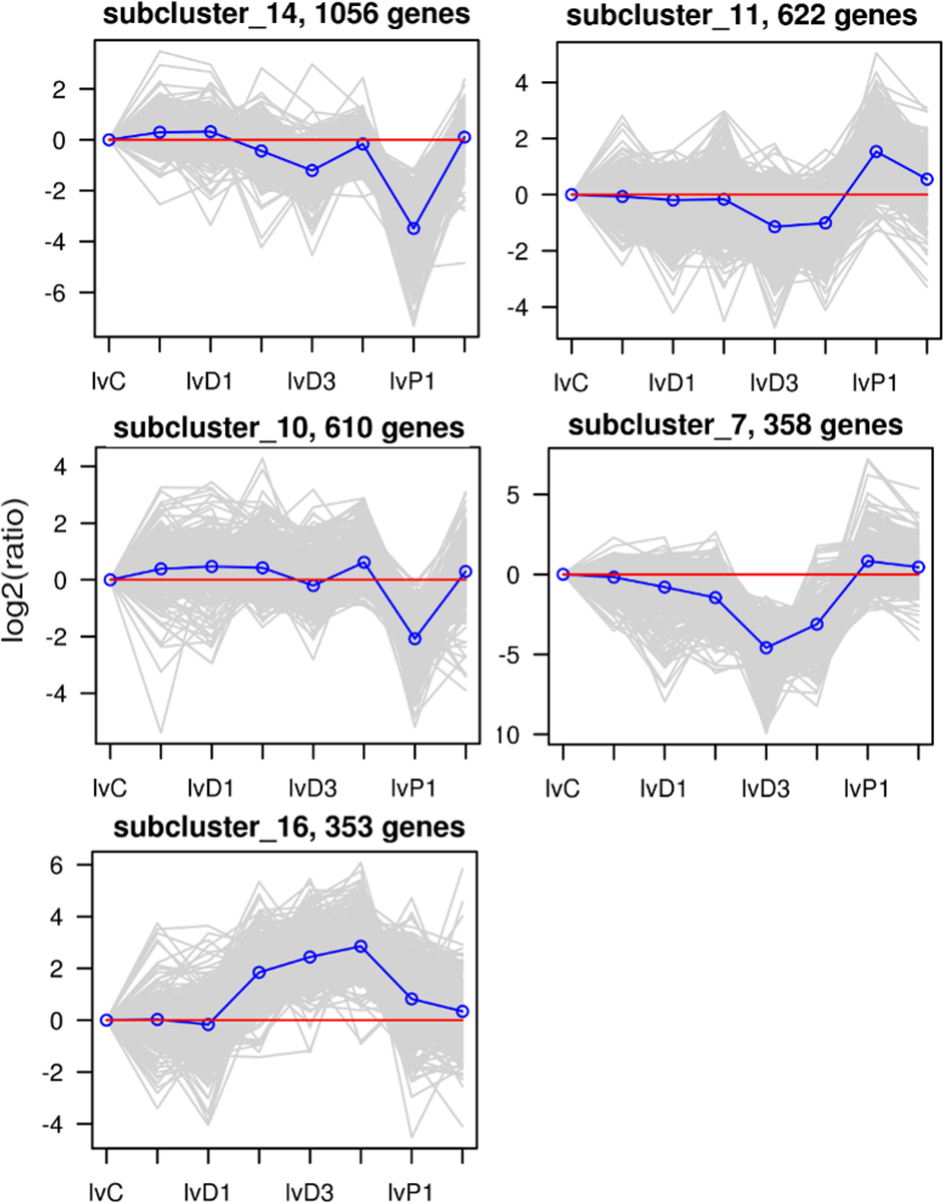
The top five clusters determined by K-means clustering, including 2,999 DEGs (58.6% of all genes). The x-axis indicates the molting stage. The y-axis indicates the log_2_(ratio) of gene expression. Each grey row represents the relative expression of DEGs in a cluster. The blue line represents the average value of all members. The red line denotes reference, the line above the red line represents up-regulation, and the line below the red line represents down-regulation. The number of DEGs within a cluster is shown after that subcluster.

Using log ratio values, we performed hierarchical clustering of DEGs expression. Expression levels during the molting cycle were divided into twenty-four categories based on K-means clustering. Detailed expression profile clusters during the eight stages are shown in S1 Fig. The largest group, subcluster 14, contains 1,056 DEGs with gene expression levels decreasing during the D2-D3 and D4-P1 transitions. Other subclusters containing over 300 members were subcluster 11 (622 DEGs), subcluster 10 (610 DEGs), subcluster 7 (358 DEGs), and subcluster 16 (353 DEGs) (Fig 3). These expression patterns not only indicate the diverse and complex interactions among genes, but also suggest that unigenes with similar expression patterns may have similar functions in the molting cycle.

### Gene ontology (GO) analysis of RNA-seq data

**Fig 4:**
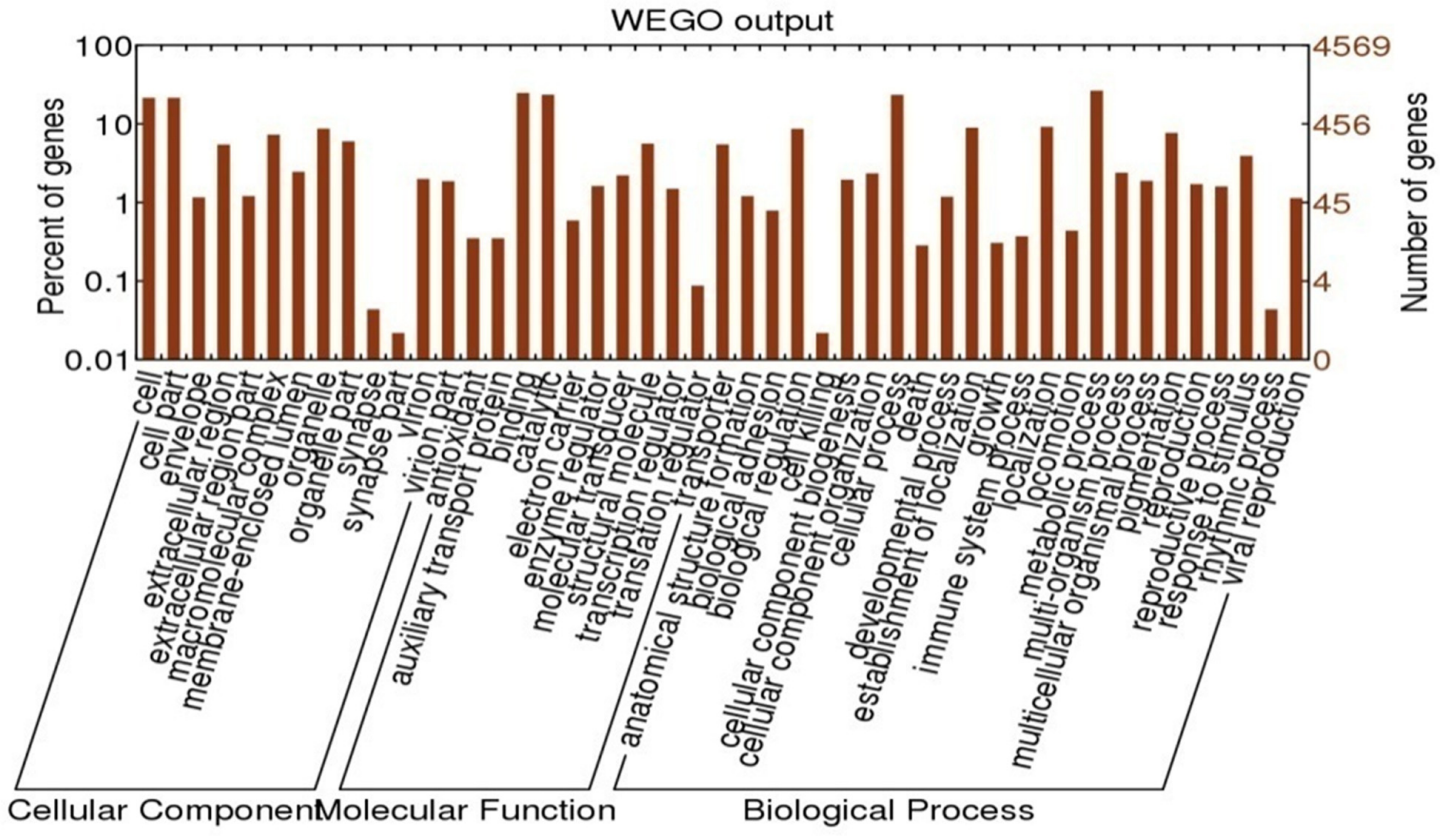
Gene ontology (GO) classification of all DEGs. The results are summarized in three main GO categories (cellular component, molecular function, and biological process). The x-axis represents GO subcategories. The left y-axis indicates the percentage of genes. The right y-axis indicates the number of DEGs.

To associate genes exhibiting different expression patterns with morphological and physiological changes during the molting cycle, we performed gene ontology (GO) enrichment analysis using BLAST2GO. A total of 11,501 GO terms were associated with all DEGs. According to the secondary classification of the GO terms, these DEGs were sorted into 47 functional groups that belong to three main GO categories: biological processes, cellular components, and molecular functions, comprised of 4,885 (42.5%), 3,606 (31.4%), and 3,010 (26.1%) DEGs, respectively (Fig 4). In the three categories, the six subcategories; cell (GO:0005623), cell part (GO:0044464), binding (GO:0005488), catalytic (GO:0003824), cellular process (GO:0009987) and metabolic process (GO:0008152) were included in the top six most-abundant sub-groups. Moreover, the major sub-categories together with the enrichment of DEGs (Corrected P-Value<0.05) among all molt stages were shown in S2 Fig.

### KEGG pathway analyses

Gene interactions play an important role in biological functions. To understand potential gene interactions underlying the molting cycle, all DEGs were searched against the Kyoto Encyclopedia of Genes and Genomes (KEGG) database, and subsequently assigned to 259 pathways. Their expression during eight stages is detailed in S4 Table. In eight comparisons, 0 (C-D0), 0 (D0-D1), 20 (D1-D2), 3 (D2-D3), 6 (D3-D4), 132 (D4-P1), 104 (P1-P2), and 119 (P2-C) pathways were enriched with DEGs, respectively. Moreover, we also found that pathways are dramatically enriched in D4-P1 and P1-P2 in terms of expression pattern and GO term analysis. Thus, we summarized the variance in KEGG pathways for molting behavior (D4-P1) and post-molt (P1-P2) (Table 2). The substantial variation in genetic pathways among developmental stages during molting might be related to morphological changes in the molting process.

**Table 2:** Significantly changed Kyoto Encyclopedia of Genes and Genomes (KEGG) pathways at pre-molt (D4–P1) and post-molt stages revealed by RNA-sequencing.

		D4-P1			P1-P2	
	Top 10 of member quantity	Top 10 of up-regulated pathway	Top 10 of down-regulated pathways	Top 10 of member quantity	Top 10 of up-regulated pathways	Top 10 of down-regulated pathways
**1**	Lysosome (55)	Tight junction (65566)	Pancreatic secretion (-15672)	Lysosome (33)	Pancreatic secretion (13332)	Tight junction (-74056)
**2**	Phagosome (22)	Focal adhesion (20354)	Protein digestion and absorption (-15559)	Metabolism of xenobiotics by cytochrome P450 (16)	Protein digestion and absorption (13067)	Adrenergic signaling in cardiomyocytes (-25196)
**3**	Amino sugar and nucleotide sugar metabolism (21)	Leukocyte transendothelial migration (19990)	Neuroactive ligand-receptor interaction (-13096)	Drug metabolism—cytochrome P450 (15)	Neuroactive ligand-receptor interaction (11855)	Focal adhesion (-24518)
**4**	Glutathione metabolism (20)	Regulation of actin cytoskeleton (19556)	Lysosome (-10954)	Amino sugar and nucleotide sugar metabolism (15)	Complement and coagulation cascades (9913)	Leukocyte transendothelial migration (-23530)
**5**	Starch and sucrose metabolism (18)	Adrenergic signaling in cardiomyocytes (18211)	Complement and coagulation cascades (-10834)	Glutathione metabolism (14)	Lysosome (9024)	Regulation of actin cytoskeleton (-23032)
**6**	Peroxisome (17)	Cardiac muscle contraction (16315)	Antigen processing and presentation (-7150)	Starch and sucrose metabolism (13)	Ribosome (7665)	Cardiac muscle contraction (-22476)
**7**	Other glycan degradation(16)	Phototransduction- fly (13935)	Ribosome (-6336)	Sphingolipid metabolism (11)	Amino sugar and nucleotide sugar metabolism (5726)	Phototransduction–fly (-14495)
**8**	Carbon metabolism (16)	Oxytocin signaling pathway (13035)	Oxidative phosphorylation (-4876)	Peroxisome (11)	Antigen processing and presentation (5712)	Oxytocin signaling pathway (-14393)
**9**	Galactose metabolism (15)	Rap1 signaling pathway (12426)	Amino sugar and nucleotide sugar metabolism (-4854)	Galactose metabolism (10)	Oxidative phosphorylation (3876)	Rap1 signaling pathway (-13989)
**10**	Glycerolipid metabolism (14)	Adherens junction (12098)	Carbohydrate digestion and absorption (-4516)	Arachidonic acid metabolism (10) Biosynthesis of amino acids (10)	Protein processing in endoplasmic reticulum (2354)	Adherens junction (-13081)

The top 10 up-regulating pathways and top 10 down-regulating pathways were identified by subtracting the fragments per kilobase of exon per million fragments mapped (FPKM) of D4 from P1 (P1 − D4) and P1 from P2 (P2 − P1). Digits in brackets indicate the number of differentially expressed genes (DEGs) and conversion of FPKM.

### Expression pattern of hormone regulation genes

Hormones have an important role in controlling the crustacean molting cycle. In our study, eleven genes related to MIH and crustacean hyperglycemic hormone (CHH) were identified. Interestingly, except for two unexpressed genes, down-regulated expressions were detected in D2-D3 in all others (Table 3). Moreover, we summarized the variance in transcript levels of molting related hormones. As a result, thirteen types of molting factors related to hormone regulation were identified. Interestingly, a trend of up-regulated transcript levels was found in all thirteen factors during D3-D4 (Table 4).

**Table 3:** Gene expression data and descriptions of 11 CHH- and MIH-related genes in the molting cycle.

Gene_id	Description	Expression							
		C	D0	D1	D2	D3	D4	P1	P2
**c104285_g1**	*L*.*vannamei molt inhibiting hormone (MIH)* gene	0	0.36	0.46	**0.94**	**0.38**	0	0	0.31
**c52158_g1**	*Lv*-MIH1 precursor	1.11	0.26	1.85	**1.14**	**0**	1.05	1.36	1.60
**c24194_g1**	*Lv*-MIH1 precursor	0.25	0	0.30	0	0	0	0.39	0.41
**c40493_g1**	molt-inhibiting hormone 1	0	0.38	0	**0.63**	**0**	0	1.59	0.66
**c65262_g1**	*L*.*vannamei* hyperglycemic hormone (CHH) mRNA	0.86	1.22	1.86	**0.95**	**0**	0.17	0.88	0.63
**c65241_g1**	hyperglycemic hormone-like peptide precursor	3.36	2.53	2.14	**25.47**	**8.43**	14.33	5.31	2.98
**c81385_g1**	Crustacean hyperglycemic hormone precursor	6.56	4.26	3.63	**6.88**	**4.23**	4.38	11.61	3.85
**c58264_g1**	Crustacean hyperglycemic hormones 4	0.47	0.21	0.94	**0.38**	**0**	0	0.68	0.58
**c64907_g1**	Crustacean hyperglycemic hormone-like peptide precursor	0.71	1.15	1.15	**3.26**	**0.47**	1.70	4.99	2.93
**c75623_g1**	Crustacean hyperglycemic hormones 2	6.01	3.66	3.24	**4.86**	**0.29**	3.72	7.05	3.68
**c72475_g2**	Crustacean hyperglycemic hormone 3 precursor	0	0	0.34	0	0	0	0	0.12

Expressions levels are represented by fragments per kilobase of exon per million fragments mapped (FPKM) at each stage. Bold text denotes down-regulation at D2 and D3. Changes in c65241_g1 expression between D2 and D3 meet the criterion of differentially expressed genes (log2ratio ≥1 and FDR ≤0.001).

**Table 4:** Expression levels of hormone related molting genes.

Factor name	Gene Number	Expression								Gene ID	Reference
		C	D0	D1	D2	D3	D4	P1	P2		
Ecdysteroid regulated-like protein	6	678.42	715.77	671.63	366.40	**187.66**	**489.67**	57.31	672.91	c19570_g1 c81580_g1 c65832_g1 c72607_g1 c74657_g1 c106886_g1	Margam*et al*., 2006 [40]
Molting fluid carboxypeptidase	4	13.04	12.32	11.83	16.76	**9.34**	**38.48**	19.61	13.34	c80306_g1 c74916_g2 c69559_g1 c64320_g1	Ote *et al*., 2005 [41]
Ecdysone receptor (ECR)	2	11.6	12.48	15.19	22.34	**9.78**	**14.20**	11.35	14.66	c72141_g9 c75175_g1	Durica*et al*., 2002 [42] Seear*et al*., 2010 [19]
Ecdysone-induced protein 74EF	1	4.30	3.65	3.31	20.40	**10.96**	**11.50**	6.33	4.70	c70692_g1	Burtis *et al*., 1990 [43]
Ecdysteroid receptor E75	1	21.94	13.04	13.60	12.17	**98.14**	**126.99**	25.76	16.57	c76549_g1	Priya *et al*., 2010 [18]
Ecdysone-induced protein 78	1	0.28	0.31	0.23	0.60	**1.37**	**2.68**	0.37	0.35	c78631_g1	Wu *et al*., 2008 [44]
Retinoid-X receptors (RXR)	2	8.95	9.09	10.42	10.34	**6.46**	**9.33**	18.01	13.03	c74810_g1 c74810_g2	Priya *et al*., 2009 [17] Durica*et al*., 2014 [45]
Methyl farnesoate(MF)	1	15.79	12.15	10.02	8.51	**2.13**	**3.66**	13.51	23.06	c40589_g1	Borst *et al*., .1987 [46]. Nagaraju 2007 [47]
Farnesoic acid (FA)	2	459.21	478.11	335.73	300.28	**267.66**	**374.55**	154.61	412.46	c69785_g1 c75001_g2	Li *et al*., .2013 [48]
Vitellogenin gene (VG)	5	52.00	126.24	99.32	34.45	**3.32**	**52.67**	29.21	55.32	c129870_g1 c47984_g2 c72701_g1 c79730_g7 c83835_g1	Shechter *et al*., 2005 [49]
17-beta-dehydrogenase	1	31.60	21.34	27.54	19.91	**3.93**	**12.51**	3.24	32.56	c71230_g1	Mindnich *et al*., 2004 [50]
Pigment dispersing hormone (PDH)	1	3.93	2.97	3.18	3.66	**3.58**	**4.35**	4.39	2.70	c82344_g1	Christie *et al*., 2014 [51]
Red pigment-concentrating hormone (RPCH)	1	4.99	5.28	2.81	4.96	**0.17**	**4.33**	7.11	3.11	c37749_g1	Sathapondecha*et al*., 2014 [52]

Molting hormone-related factors and fragments per kilobase of exon per million fragments mapped (FPKM) are shown for each stage. Expression represents the FPKM value of combined factors. Bold text denotes up-regulated expression in D3 and D4. The reports about genes related to molting hormone were used as the reference.

### Expression profile of skelemin genes

Expression analysis revealed high transcript levels of myosin, troponin, and actin following molting behavior (D4-P1). Members with high expression (FPKM value >1000 in at least one stage) were grouped together, and 49 members were found (6 were actin, 39 were myosin and 4 were troponin) (Table 5). Interestingly, 98% of the factors showed an up-regulated expression in D4-P1 (Table 5 bolded text). Some factors showed large changes in expression. For example, the FPKM values of actin T2 (c82047_g1) and skeletal muscle actin 6 (c78558_g1) increased more than 11,000 at D4-P1. Additionally, the FPKM values of myosin light chain (c26953_g1) and myosin light chain 2 (c61912_g2) increased more than 7,800, and those of troponin T (c70761_g2 and c59125_g1) increased more than 3,300 during molting.

**Table 5:** Expression levels of actin, myosin and troponin genes.

gene_id	Expression								Description
	C	D0	D1	D2	D3	D4	P1	P2	
**Actin**									
c82047_g1	20849.17	11760.99	8767.04	17445.72	37490.78	**14821.08**	**27282.26**	12889.42	actin T2 [*Litopenaeus vannamei*]
c78558_g1	19381.33	12253.83	11127.16	15866.74	29958.74	**13252.85**	**24546.41**	13282.95	skeletal muscle actin 6 [*Homarus americanus*]
c82047_g2	5452.26	4508.13	3889.94	5610.92	7692.16	**5088.78**	**5698.87**	4280.14	actin [*Hypochilus thorelli*]
c1146_g1	551.34	356.10	353.85	522.41	1019.68	**482.48**	**815.53**	442.82	PREDICTED: similar to alpha actinin CG4376-PB [*Tribolium castaneum*]
c64985_g1	582.23	285.86	189.72	431.65	1147.69	**409.86**	**573.96**	349.84	beta-actin [*Scylla paramamosain*]
c67471_g1	5305.29	5754.25	4079.25	5732.58	4737.16	5551.05	4644.41	5148.22	actin E [*Litopenaeus vannamei*]
**Myosin**									
c26953_g1	18358.27	9826.73	8751.77	13851.15	30821.99	**13627.39**	**21918.84**	11310.27	myosin light chain [*Marsupenaeus japonicus*]
c61912_g2	17348.02	8694.49	5830.68	12411.85	29644.34	**12465.05**	**20273.25**	10038.46	myosin light chain 2 [*Procambarus clarkii*]
c83685_g5	6013.84	3319.11	3139.47	5724.11	10316.04	**4682.86**	**8209.14**	4013.95	myosin heavy chain type a [*Marsupenaeus japonicus*]
c83685_g6	5284.95	3459.07	2954.50	4903.49	9770.18	**4029.98**	**6860.66**	3898.41	myosin heavy chain [*Farfantepenaeus paulensis*]
c83685_g2	5493.96	3169.90	2752.71	4762.17	10266.71	**4064.44**	**6762.04**	3497.44	myosin heavy chain type 2 [*Penaeus monodon*]
c71354_g2	5380.86	3220.31	2368.17	4408.20	8691.22	**4199.79**	**6297.09**	3648.19	Tropomyosin
c59746_g1	4283.22	2365.53	2251.28	3796.17	8245.85	**3350.06**	**5367.31**	2700.07	myosin heavy chain type b [*Marsupenaeus japonicus*]
c83685_g3	3713.36	1950.47	1416.29	3185.80	6668.01	**2855.43**	**4750.30**	2330.42	myosin heavy chain type a [*Marsupenaeus japonicus*]
c83685_g7	3350.25	1845.41	1403.93	3129.62	6009.07	**2616.69**	**4487.76**	2277.59	myosin heavy chain type 1 [*Penaeus monodon*]
c80445_g4	2002.07	1569.03	1140.62	2575.80	2775.48	**1849.93**	**3697.77**	2244.00	myosin heavy chain type a [*Marsupenaeus japonicus*]
c73388_g1	3341.68	1750.24	1518.60	2894.58	5650.37	**2522.68**	**4290.25**	2141.69	myosin heavy chain type 1 [*Litopenaeus vannamei*]
c72756_g3	3191.01	1910.18	1279.89	3158.56	5343.45	**2612.57**	**4215.41**	2172.07	myosin heavy chain type b [*Marsupenaeus japonicus*]
c83685_g1	2957.29	1762.68	1486.49	2904.02	5242.33	**2381.47**	**3902.89**	1990.97	myosin heavy chain type 2 [*Litopenaeus vannamei*]
c56242_g1	1817.63	1214.37	817.26	1998.79	2457.60	**1438.90**	**2919.95**	1696.15	myosin heavy chain [*Farfantepenaeus paulensis*]
c83685_g4	2487.71	1667.46	1287.52	2516.83	4704.41	**1959.07**	**3307.62**	1842.34	myosin heavy chain type 2 [*Litopenaeus vannamei*]
c28182_g1	1241.64	957.46	468.61	2004.75	1439.54	**1270.13**	**2572.92**	1532.49	myosin heavy chain type 1 [*Litopenaeus vannamei*]
c70174_g1	1293.27	989.18	687.60	1728.27	1600.99	**1204.38**	**2383.42**	1419.61	myosin heavy chain type a [*Marsupenaeus japonicus*]
c81295_g1	2174.39	1454.10	1238.15	2324.49	3751.85	**1746.31**	**2886.82**	1608.96	myosin heavy chain type 2 [*Litopenaeus vannamei*]
c72756_g5	1906.57	1181.00	855.85	2022.35	3457.55	**1658.71**	**2737.90**	1386.27	myosin heavy chain type 2 [*Penaeus monodon*]
c80636_g6	1173.97	834.21	622.22	1483.74	1867.45	**1047.43**	**1914.98**	1055.14	myosin heavy chain type a [*Marsupenaeus japonicus*]
c73679_g4	1287.09	1064.99	891.33	1744.40	2259.05	**1153.50**	**2002.83**	1179.03	myosin heavy chain type 2 [*Penaeus monodon*]
c75308_g3	1160.26	763.11	581.75	1150.50	2075.74	**898.38**	**1710.23**	939.38	myosin heavy chain [*Farfantepenaeus paulensis*]
c65216_g3	762.80	614.08	496.68	1087.12	929.43	**748.72**	**1523.21**	845.22	myosin heavy chain type 1 [*Penaeus monodon*]
c54630_g1	757.96	388.83	381.84	901.29	1239.59	**556.49**	**1166.68**	570.87	myosin heavy chain type 2 [*Litopenaeus vannamei*]
c80636_g3	606.17	465.76	292.61	874.87	637.77	**563.28**	**1168.67**	692.98	myosin heavy chain type 1 [*Litopenaeus vannamei*]
c67474_g3	658.03	537.14	522.17	708.15	885.97	**578.37**	**1151.96**	547.11	myosin heavy chain type b [*Marsupenaeus japonicus*]
c131544_g1	1001.89	456.89	474.76	748.11	1719.62	**658.40**	**1207.93**	506.46	myosin heavy chain type 1 [*Penaeus monodon*]
c76934_g4	241.76	231.68	124.32	237.08	148.35	**170.21**	**709.00**	343.57	myosin light chain [*Penaeus monodon*]
c80445_g3	1158.72	660.06	569.76	983.21	2504.93	**890.92**	**1403.62**	727.07	myosin heavy chain type a [*Marsupenaeus japonicus*]
c61703_g1	577.82	370.49	161.21	639.87	840.82	**507.30**	**1014.06**	496.98	myosin heavy chain type 1 [*Litopenaeus vannamei*]
c56973_g1	723.62	583.59	743.88	622.63	1086.04	**512.81**	**977.64**	588.23	myosin heavy chain type a [*Marsupenaeus japonicus*]
c26953_g2	15234.89	7968.36	5883.06	11377.27	25713.28	**11059.43**	**18522.19**	9214.75	*Marsupenaeus japonicus* myosin light chain mRNA
c60870_g1	457.39	207.31	203.58	266.65	1093.96	**283.22**	**486.43**	176.09	myosin heavy chain type 1 [*Penaeus monodon*]
c49254_g1	461.37	176.57	179.47	261.17	1069.88	**289.38**	**389.47**	168.66	myosin heavy chain type a [*Marsupenaeus japonicus*]
c44371_g1	800.12	582.47	469.72	675.81	1261.00	**657.07**	**1080.53**	610.90	paramyosin [*Papilio xuthus*]
c75308_g4	652.67	368.88	264.29	572.09	1413.10	**537.57**	**838.36**	469.15	myosin heavy chain [*Farfantepenaeus paulensis*]
c44674_g2	617.30	446.94	628.45	518.82	1321.63	**443.16**	**740.85**	410.36	muscle myosin heavy chain [*Papilio xuthus*]
c73679_g12	679.31	360.95	342.99	551.67	1214.87	**506.63**	**784.06**	405.63	myosin heavy chain type 2 [*Penaeus monodon*]
c54360_g1	686.35	447.84	349.10	491.94	1021.10	**527.31**	**822.92**	468.13	myosin heavy chain isoform 3 [*Ocypode quadrata*]
**Troponin**									
c70761_g2	7319.28	4474.64	3958.16	6386.01	12737.54	**5811.09**	**9338.42**	5377.96	Troponin T [*Lepeophtheirus salmonis*]
c59125_g1	7150.50	3794.77	3426.53	5099.38	10466.81	**4802.50**	**8132.46**	4293.31	troponin I [*Litopenaeus vannamei*]
c81020_g2	1656.78	878.59	824.72	1419.39	1964.06	**1214.66**	**2228.47**	1118.01	troponin C1 [*Litopenaeus vannamei*]
c81020_g3	800.81	349.56	280.64	353.36	1150.26	**534.82**	**582.99**	321.51	troponin C [*Penaeus monodon*]

Skelemin related factors and fragments per kilobase of exon per million fragments mapped (FPKM) are shown for each stage. Bolded text denotes up-regulated expression in D4-P1.

### Expression pattern of immune related genes

**Fig 5:**
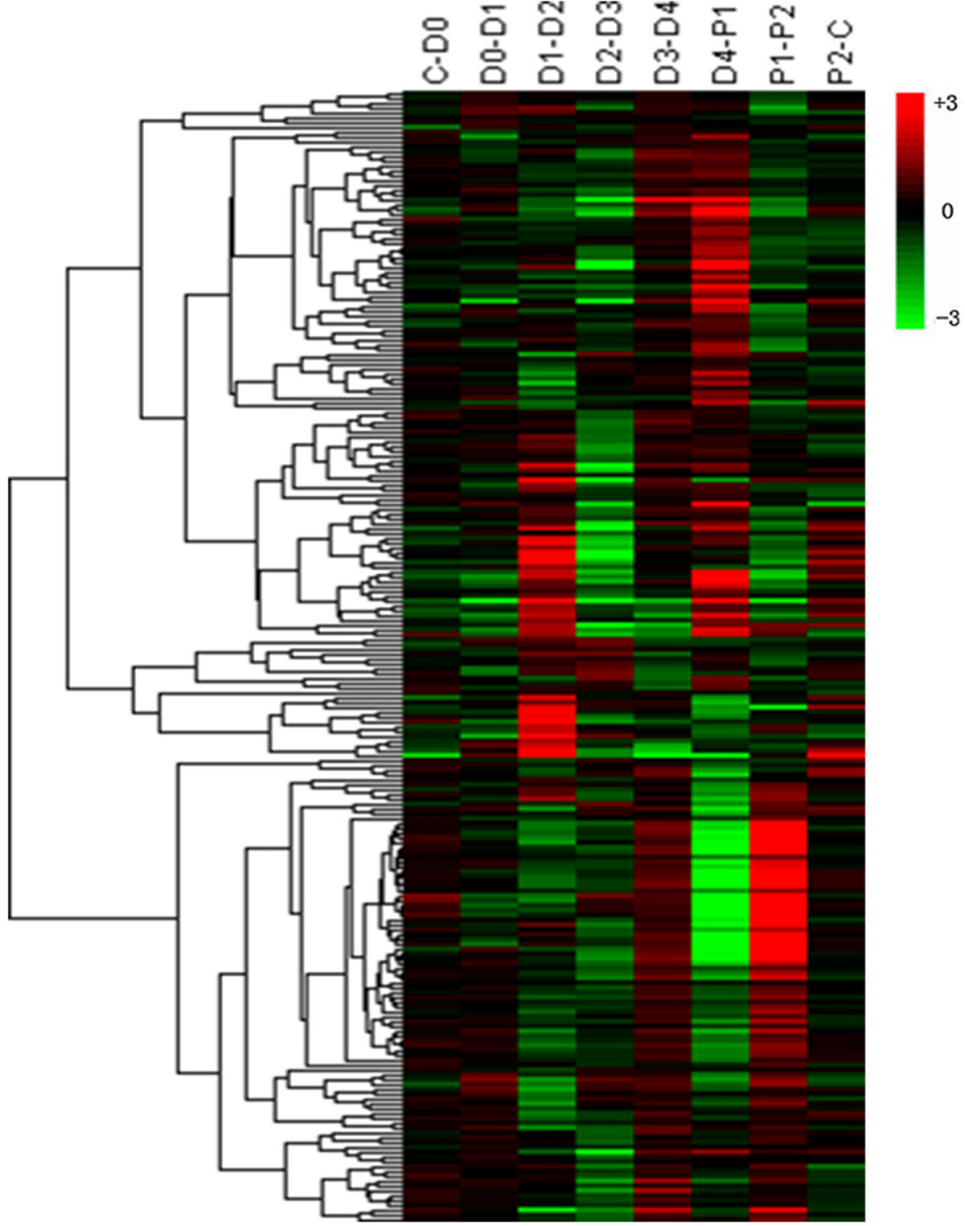
Hierarchical clustering of 236 immunity genes. Colored keys represent the fold changes (log_2_ transformed counts) of gene expression between adjacent molt stages Red represents up-regulation and green represents down-regulation. Each column represents an experimental condition (e.g. C-D0), and each row represents an immunity gene.

To reveal the expression changes of the immune-related genes during molting, we summed the expression patterns of the immune pathways as a proxy for the immunity level of shrimp according to Li and Xiang (2013a,b) [53,54]. Thirty-six immunity families, including 236 immune genes, were categorized between molting stages. Clusters displayed expression patterns for a subset of all immunity genes in each comparison (Fig 5). Interestingly, most immune factors were up-regulated in D3-D4, including thirty-two clusters (32/36 clusters, 81.8% of all 236 genes). During molting behavior (D4-P1), some immune factors were up-regulated, including 16 cluster factors, and the majority was consist of crustin and anti-lipopolysaccharide factor (ALF), which demonstrated expression (FPKM value) level increases of 7.6 and 3.3 folds, respectively, after ecdysis.

### Expression changes of hemocyanin, chitinase and serine protease superfamily in molt process

**Fig 6:**
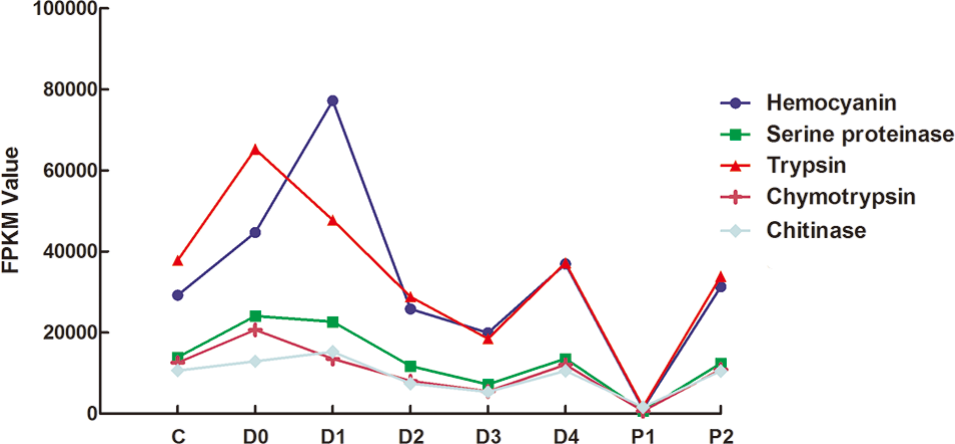
Expression profiles of hemocyanin, serine proteinase, trypsin, chymotrypsin, and chitinase. The x-axis represents developmental stages. The line represents the total fragments per kilobase of exon per million fragments mapped (FPKM) for the genes.

To identify the possible factors involved in the molting process, we investigated the expression profile of members of the hemocyanin, chitinase and serine protease superfamily during the molting process. Data mining of the annotated genes identified twelve genes associated with hemocyanin. Similarly, 19, 16, and five genes were found to be related to serine proteinase, trypsin, and chymotrypsin, respectively. Twenty-three unigenes related to chitinase were identified in this study. The expression profile of hemocyanin genes showed a characteristic pattern of up-regulation at inter-molt and down-regulation during D1-D3. In D3-D4, expression was up-regulated, and then declined to the minimum in P1 (Fig 6). Surprisingly, the expression profile of hemocyanin and chitinase were consistent. The expression data revealed that genes corresponding to chitinase were preferentially expressed during periods D1 and D4. Moreover, similar expression patterns were observed in three serine protease superfamily members, including serine protease, trypsin, and chymotrypsin. Serine protease, trypsin, and chymotrypsin displayed expression changes similar to hemocyanin and chitinase, up-regulated in C-D0, D3-D4, and P1-P2 (Fig 6).

### Real-time quantitative PCR validation of RNA-seq results

**Fig 7:**
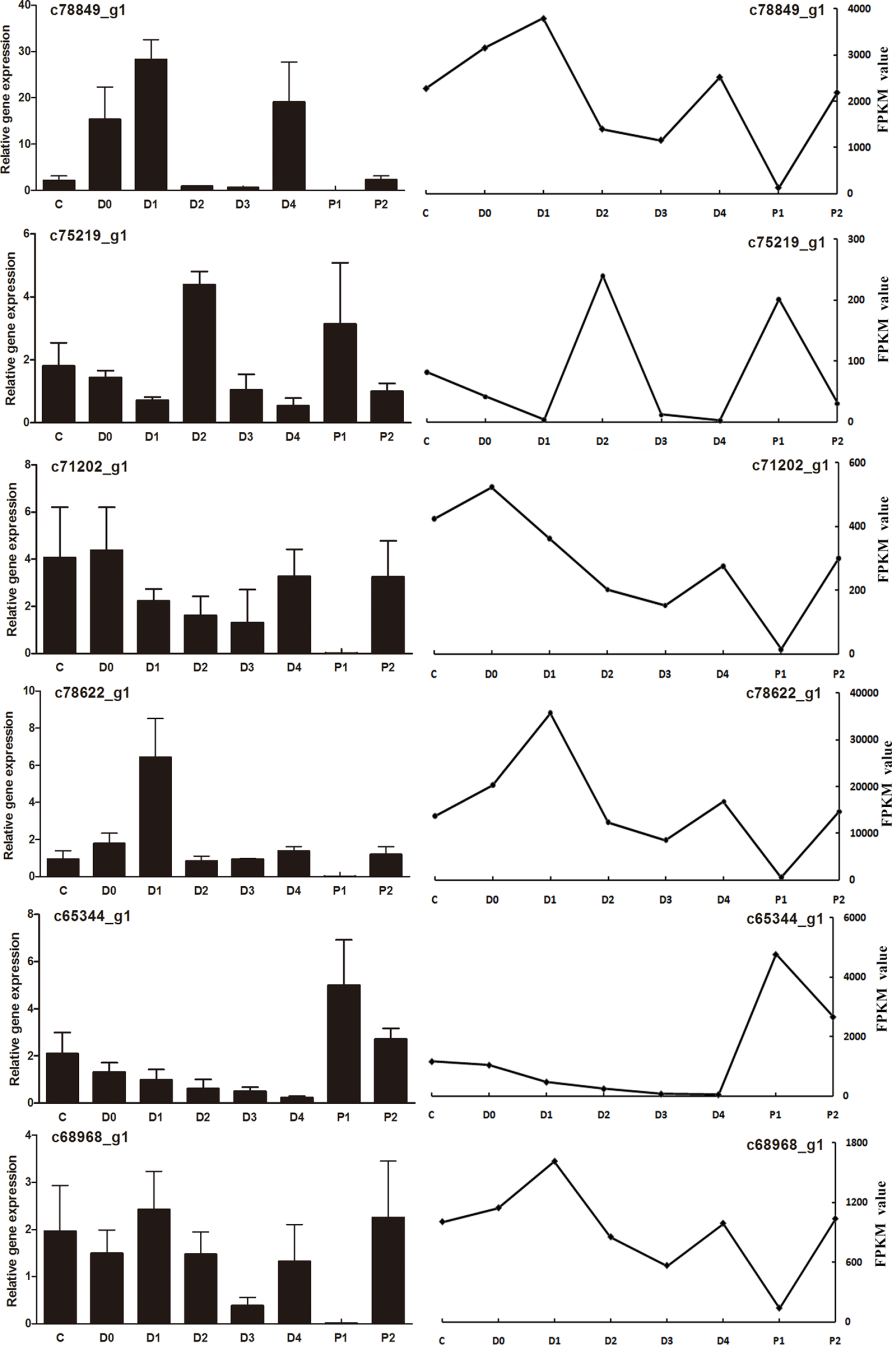
Real-time quantitative PCR (qRT-PCR). Six candidate genes were selected randomly for validation. Expression results of qRT-PCR are shown on the left and expression profiles of RNA-sequencing on the right. The gene c82047_g1 (*Actin T2*) was used as the control reference gene.

To validate sequencing data, six differentially expressed genes were selected for real-time qPCR analysis. Expression patterns of the selected genes determined by real-time qPCR (Fig 7) are consistent with those determined by RNA-seq, corroborating our results.

## Discussion

### Establishment of an overall transcriptome database for the molting process

The results of the transcriptome analysis presented here establish a genome-wide transcriptional landscape for further studies on the developmental and molecular aspects of the molting process in *L*. *vannamei*. In terms of RNA-seq results, it was found that the expression changed markedly in D4-P1 and P1-P2. It is known that molting behavior occurs in D4-P1, while P1-P2 is the recovery stage [2]. There is an obvious morphological diversity between these stages. However, the molecular mechanisms involved in molting behavior are not fully elucidated. Our RNA-seq results provide useful insights into the molecular processes underlying these behaviors.

By profiling gene expressions and expression changes, it was found that down-regulated genes were predominant in D4-P1, while in P1-P2, various genes were mainly up-regulated (Fig 2). Based on this result, the DEGs enriched in GO terms related to the shrimp metabolism level were significantly down-regulated during D4-P1. The top five down-regulated terms were GO:0009987 “cellular process” (294 down-regulated DEGs), GO:0008152 “metabolic process” (288 down-regulated DEGs), GO:0005488 “binding” (276 down-regulated DEGs), GO:0071704 “organic substance metabolic process” (249 down-regulated DEGs), and GO:0003824 “catalytic activity” (232 down-regulated DEGs). While the GO terms in P1-P2 were majorly up-regulated, the terms which showed down-regulation in D4-P1 were up-regulated back. Moreover, DEGs for pancreatic secretion (map04972), protein digestion and absorption (map04974), neuroactive ligand-receptor interaction (map04080), lysosome (map04142), and other pathways were down-regulated during D4-P1. In P1-P2, however, protein digestion and absorption, neuroactive ligand-receptor interaction, and lysosome-related pathways were recovered (up-regulated). These results were consistent with the morphological changes and the variation in molecular changes between molting behavior and post-molt. In summary, the RNA-seq results provide the basal understanding for the molecular changes occurring during the molting process.

### Hormone regulation process in *L*. *vannamei* molting

It is now well established that MIH and CHH peptide families serve as key regulators of hormones controlling the molting process [55]. MIH, which is secreted from a neuro-secretory center termed the X-organ/sinus gland complex (XO/SG) in crustacean eyestalks, is a member of the CHH family [13,56]. Extirpation of XO/SG can reduce MIH secretion, enable ecdysteroid synthesis, and improve molt frequency [57]. In our study, down-regulated expressions of all MIH and CHH were detected in D2-D3 (Table 3). Based on the inhibitory effect of MIH and CHH on ecdysone secretion, this result suggests that down-regulation of MIH and CHH-related genes may trigger the molting process, and D2-D3 likely represents the onset of molt hormone-regulation. This speculation is supported further by physiological evidence that reduction in sinus gland MIH content occurs during the late pre-molt stage in *Procambarus clarkia* [58].The concentration of MIH was found to decrease in pre-molt, followed by an increase in ecdysone in the hemolymph [55]. Furthermore, qualitative measurements of MIH mRNA transcript abundance found MIH peptide synthesis to be dramatically reduced during late pre-molt in eyestalks of *Callinectes sapidus* [59].

Only few studies have focused on the molecular role of molting-related hormones followed by MIH and CHH. To investigate hormones downstream of MIH and CHH in the molting process pathway and elucidate possible roles in this process, we summarized the variance in transcript levels of molting related hormones. According to the RNA-seq results, a trend of up-regulated transcript levels was found in all thirteen factors during D3-D4 (Table 4). Most of these factors were reported previously to be associated with molting; for example, ecdysone receptors (ECRs) and retinoid-X receptors (RXRs) have been found to be potential targets for hormonal control during limb regeneration and molting in *Uca pugilator* [45]. In this study, we also identified two ECRs genes (c72141_g9 and c75175_g1) and two RXRs genes (c74810_g1 and c74810_g2). Methyl farnesoate (MF) and farnesoic acid (FA), secreted by the mandibular organs (MO), might have specific stimulatory effects on Y-organs where ecdysteroids are secreted [47]. Synthesis of the vitellogenin gene (*VG*) in *Cherax quadricarinatus* has been shown to be induced by 20E, it has been suggested that *VG* is an ecdysteroid-responsive gene in the molt process [49]. In this study, a trend of up-regulated transcript levels was found in all twelve factors during D3-D4 (Table 4). In light of the fact that the D3-D4 stage occurs immediately before ecdysis, this result suggests that these hormones are associated with molting during the D3-D4 stage. Indeed, D3-D4 likely serves as the implementation phase for molt hormone-regulation. Moreover, the presence of specific up-regulated genes in the molt phase indicates that they could participate in hormone regulation and promote the molting process, as seen in three ecdysteroid-regulated genes (c19570_g1, c81580_g1, and c65832_g1) whose expression increased 2.31, 2.82, and 3.45 fold during D3 to D4, respectively. Similarly, two molting fluid carboxy peptidase precursors (c80306_g1 and c74916_g2) increased 3.21 and 24.5 fold in D3-D4. These genes, potentially involved in hormone regulation, will serve as promising candidates for future analyses.

In summary, we infer that hormone-regulated molting signals likely play distinctly different roles in shrimp molting. Our findings show the first step of molting to be the down-regulation of MIH and CHH family peptides in D2-D3 to initiate the process. Once inhibition by MIH and CHH is prevented, downstream hormones and other factors can participate in regulation in the D3-D4 stage of molting.

### Analysis of skelemin in molting process

Post-molt is a period of rapid enlargement and growth for shrimp as a result of body size expansion and exoskeleton reconstruction after ecdysis. The process is complex, involving not just fast water absorption, but also expansion of the skelemin to provide a scaffold and muscle to fill the new body [60]. Skelemin is important for body reconstruction after molting [61]. However, factors participating in enlargement as skelemin during the molting process have remained largely unknown. According to the RNA-seq results, genes related to actin, myosin and troponin were markedly up-regulated after molting (D4-P1) (Table 5). Actin is a globular multi-functional protein that forms microfilaments and serves as the major component of muscle [62]. It is reported that the transcriptional response of actin (*PotActinSK1*, *PotActinSK2*, *PotActinHT*, and *PotActinCT*) to 20-hydroxyecdysone (20E) injection was different in *Portunus trituberculatus*, suggesting that 20E affects muscle plasticity [63]. Myosin is the major component of thick filaments in muscle, and troponin is a complex of three regulatory proteins (troponin C, troponin I, and troponin T) integral to muscle contraction in skeletal and cardiac muscle [64]. As the major contractile protein in vertebrates and invertebrates, myosin was identified to be associated with muscle atrophy during molting, which was characterized by decrease in fiber width and myofibril cross-sectional areas, increase in inter fibrillar spaces, and degradation [65,66]. Troponin and tropomyosin are modulatory proteins that modulate the binding of actin to myosin [67]. Indeed, body enlargement after molting requires frames and skelemin. Considering the expression profiles of actin, myosin, and troponin after molting (Table 5), our findings suggest that these three proteins are expressed abundantly along with skelemin to support the new body of the shrimp after the phase of molting behavior is complete.

### Immunity tactic during molting cycle in *L*. *vannamei*

Immediately following ecdysis, the new exoskeleton is soft and the exercise capacity of shrimp is very weak, so post-molt (P1) is the most sensitive period to suffer an attack from bacteria, viruses, or other predation. It is known that shrimp practice cannibalism which can abet the outbreak of infectious diseases such as White Spot Syndrome (WSS) [68] and the targets of cannibalism are mainly post-molt shrimp. The immune and protection tactics that allow post-molt shrimp to avoid stress are unknown. To address questions regarding post-molt defense, we summed the expression patterns of the immune pathways as a proxy for the immunity level of shrimp according to Li and Xiang (2013a,b) [53,54]. Interestingly, most immune factors were up-regulated in D3-D4. And during molting behavior (D4-P1), parts of immune factors were up-regulated, and the majority up-regulated factors were concentrated in the some downstream and executive factors of immune pathway, like crustin and ALF. Invertebrates including shrimp have no real adaptive immunity and mainly depend on their innate immunity against a variety of pathogens [69]. Antimicrobial peptides (AMPs) are important effectors in innate immunity, which is the front line of host defense against infection by microbes including bacteria, fungi, and viruses [70,71]. Crustin and ALF are important AMPs that play an important role in the immunity of shrimp [72]. Crustin (*MjCru I-1*) found in *Marsupenaeus japonicus*can bind to the cell wall molecules of bacteria, such as lipopolysaccharide (LPS), peptidoglycan (PGN), and lipoteichoic acid (LTA) [69]. *LvALF* from *Litopenaeus vannamei*, *MjALF* from *Marsupenaeus japonicus*, *PtALF* from *Portunus trituberculatus*, and *EsALF* from *Eriocheir sinensis* have also been reported to show broad-spectrum activity against gram-positive and gram-negative bacteria, fungi, and viruses [73–76].

In summary, we propose that shrimp will up-regulate the expression of immune-related genes initially in D3-D4. After ecdysis, several downstream factors, such as the antimicrobial peptides, and other factors, such as the i-type lysozyme-like protein 2 (c72940_g1) that is up-regulated about 25-folds between D4 and P1, are likely to be expressed to generate an immune response. Once the exoskeleton hardens, the shrimp acquire new protection, and the expression of immune genes recovers to the baseline level.

### Expression changes of hemocyanin, chitinase and serine protease superfamily in molt process

Some factors have been reported to be associated with the molting process in previous studies. It reported that hemocyanin highly expressed in both the inter-molt and pre-molt periods is reflective of the dual functionality of hemocyanin in preparation for arthropod ecdysis in *Portunus pelagicus* [77]. Studies have also confirmed that the molting process can affect hemocyanin levels in *L*. *vannamei* juveniles [78]. Chitinase is the main enzyme hydrolyzing the glycosidic bonds in chitin to digest old exoskeleton partially [79]. The structures of serine protease superfamily are very similar, even though they recognize different substrates [80–82]. The serine protease superfamily members and hemocyanin share phenoloxidase (PO) activity, and studies on *L*. *vannamei* have suggested the regulation of trypsin biosynthesis may be, at least in part, under the influence of ecdysteroid hormones [83]. Hence, in our study, expression of serine protease superfamily members correlates strongly with chitinase expression. Taken together with the functional relatedness of these enzymes, this result suggests a shared or similar function in degradation or digestion in the molting cycle. Moreover, the similarity in gene expression profiles of hemocyanin and these three members of the serine protease superfamily may be indicative of activation of the PO pathway. In this study, the RNA-seq results also illustrated that these factors might be associated with molting in *L*. *vannamei*.

### Other factors that may be associated with the molting process

Aquaporins (AQPs) are essential to facilitate the transport of water and other small polar molecules across cell membranes and play an important role in osmotic regulation [84]. After their escape from the confines of a cuticle, shrimp need to rapidly take up water [2]. One aquaporin gene, c76592_g1, was identified in the molting transcriptome and its expression level was the highest after molting behavior (P1), which indicated that it played a role in osmotic regulation during the molting process.

The formation of chitin is catalyzed by chitin synthetase, a highly conserved enzyme involved in chitin synthesis [79]. One chitin synthetase gene (c79847_g1) was found in the transcriptome result; expression analysis showed that it reached a peak in D4 and P2. Along with its functional annotation, chitin synthetase has been suggested to be involved in new cuticle synthesis in D4 and P2.

Myostatin (*MSTN/GDF11*) was identified to act as a negative regulator of muscle development in vertebrates and in the shrimp *Macrobrachium nipponense*. During the molt cycle, the expression of *Mn-MSTN/GDF11* mRNA was up-regulated significantly at the early post-molt stage, but later decreased gradually [85]. We also found one myostatin gene (c72168_g1) in the molting transcriptome, and the expression pattern was similar to that in *Macrobrachium nipponense*, with an up-regulation at post-molt, followed by a down-regulation. These results indicated that myostatin may play a role in the molt cycle.

An earlier report showed that knockdown of a novel G-protein pathway suppressor 2 (*GPS2*) (GenBank accession number JN714124) led to mortality by exuvial entrapment during ecdysis in shrimp (*Penaeus vannamei*) [86]. In our study, we identified a *GPS2* gene, c74297_g2, and its expression exhibited low variance levels; this gene was thought to be a housekeeping gene that acts during the molting cycle in *L*. *vannamei*.

### Comparison with results of transcriptomic analyses in other arthropods

There have been a number of previous reports on the transcriptomic analysis of the molting process in other arthropods. By analyzing the gene expression profiles in whole animals and organs previously identified to be related to molting by microarray analysis, Kuballa and Elizur performed differential expression profiling of components associated with exoskeletal hardening in the crab *P*. *pelagicus*. Moreover, it was found that the C-type lectin receptor, mannose binding protein, PO (trypsin-like, clotting protein precursor-like and antimicrobial proteins) inducers and antimicrobial proteins, in conjunction with hemocyanin, displayed molt cycle-related differential expression profiles, which indicated that they had possible regulatory functions in the calcification or sclerotization cascade of the cuticle [87]. A microarray-based investigation was performed using mRNA collected from the larval stage to the pupal stage for analyzing the cuticular genes in the silkworm, *Bombyx mori* [88]. A total of 227 cuticular protein genes were found to be expressed, and their expression profiles and clustering was discussed. Custom cDNA microarrays were constructed for the crab *Portunus pelagicus* to analyze specific differential genes in the molt cycle [77]. By analyzing the expression profiles in thoracic exoskeleton formation during the pupal-to-adult molting in *Apis mellifera* by microarray analysis, it was found that 1,253 unique DEGs and 547 were up-regulated in the thoracic dorsum after molting, which suggested an induction of expression by the ecdysteroid pulse [89]. Furthermore, 28 of the 36 muscle-related DEGs were up-regulated during the formation of striated fibers attached to the exoskeleton. Another transcriptomic analysis focused on chitin metabolism in four stages (inter-molt, early pre-molt, late pre-molt and post-molt) in the crayfish *Cherax quadricarinatus* with regard to three key chitin metabolic processes, chitin synthesis, chitin breakdown, and the junction between the metabolism of simple sugars and amino sugars [90].

There were some similarities compared to the results of our work, for example, some common factors are considered to be related to the molt cycle, like the cuticular genes, PO family, chitin synthase, antimicrobial proteins and hemocyanin. However, several differences exist between our results and those of previous studies. These differences may be due to the difference in the techniques used (RNA-seq and microarray). The transcriptome database obtained using Illumina RNA-seq in our study was larger than that obtained by microarray-based analysis in other molting animals, and more genes were detected at each stage of the molting process in our study. Secondly, previous studies mainly focused on exoskeleton reconstruction, but hormone regulation, expression profiles during molting behavior, skelemin, and immunity tactics have not been analyzed so far. We focused on these issues and provided insights into the molecular mechanisms underlying the molting process. This is also the first time that the most important economic species of shrimp, *L*. *vannamei*, was used to analyze the transcriptome of whole animals during the molting cycle.

We obtained a considerable number of expressed genes; however, we did not investigate the expression of all genes and focused only on the highly expressed ones. Therefore, some infrequently expressed factors that play important roles in molting may be absent. This limitation can be solved by analyzing the transcriptomes of different tissues and developmental stages in shrimp [91–95] or genes with limited expression.

## Conclusion

**Fig 8:**
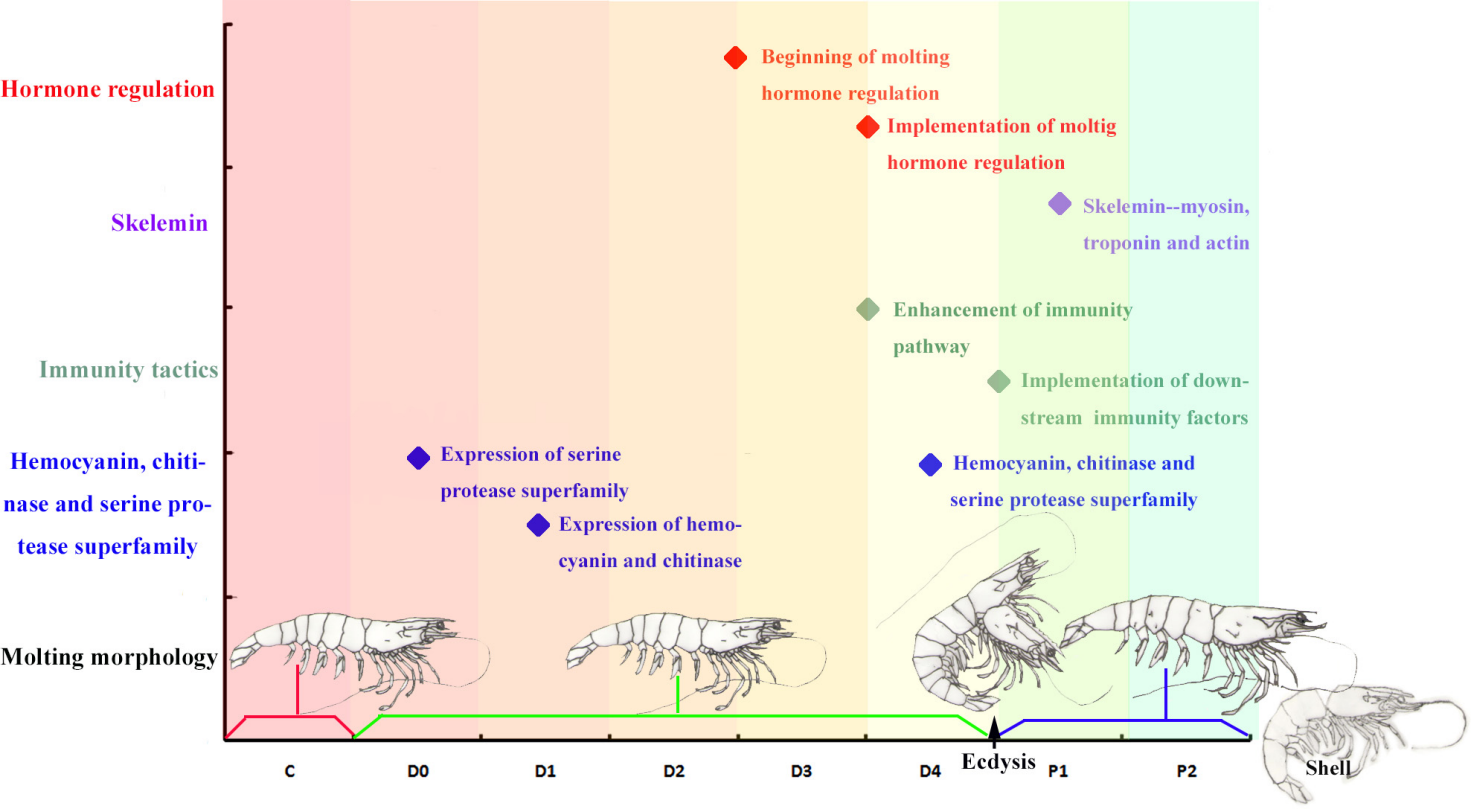
A cascade of molecular events applicable to the molting cycle of *L*. *vannamei*. This figure depicts the molecular events identified in this study in relation to the morphological diversification of molting in a diagrammatic sketch. The rhombi represent molecular events occurring in the molting process. The x-axis represents molting stages.

Here, we report the first comprehensive study utilizing RNA-seq to characterize genes associated with shrimp molting and to deduce the molecular events involved in the molting process. The results provided a basic understanding of the molecular mechanisms undergirding molting, including hormone regulation, skelemin, immune response *etc*. Our results show that molting cycles of *L*. *vannamei* are encoded by a large number of gene families subject to strict patterns of temporal and spatial regulation. We propose a cascade of molecular events applicable to the molting cycle of *L*. *vannamei* (Fig 8). These comprehensive analyses provide molecular evidence that will improve our understanding of morphological variation in molting and serve as a potential blueprint for future research on molting in crustaceans and other molting animals.

## Supporting Information

S1 Fig The twenty-four clusters including all 5,117 DEGs divided by K_means_cluster method. The x-axis indicates molting stage. The y-axis indicates the log_2_(ratio) of gene expression. Each grey row represents the relative expression of DEGs in a cluster. The blue line represents the average value of all members in one cluster. The red line indicates reference. Above the red reference line represents up-regulation, and below it represents down-regulation. The total number within a cluster is shown after each subcluster.
(JPG)

S2 Fig Gene ontology enrichment of all DEGs in eight comparisons. The results are summarized in three main GO categories. The x-axis represents the names of these GO subcategories. The left y-axis indicates the percentage of genes. The right y-axis indicates the number of DEGs expressed in a given sub-category.
(JPG)

S1 Table Oligonucleotide primers of six genes for the verification experiment. Gene descriptions, along with product lengths in brackets, are provided.
(DOCX)

S2 Table Details of 93,756 unigenes from *L*. *vannamei* molting cycle. Each row is a unigene identified from the molting transcriptome. Information provided in the table for each unigene are the expression (FPKM value) in eight stages (C, D0, D1, D2, D3, D4, P1, and P2), unigene length, NCBI non-redundant nucleotide sequence (NR) description, NCBI non-redundant nucleotide sequence (NT) description, KEGG Orthology (KO) description, Swissprot description, PFAM description, GO annotation (biological process, molecular function, and cellular component), and KOG description.
(XLSX)

S3 Table Expression profiles of all 5,117 DEGs from the *L*. *vannamei* molting cycle. Each row is a DEG identified from the molting transcriptome. Expression levels in all eight stages are provided for each unigene.
(XLSX)

S4 Table Details of 259 putative pathways identified for the eight stages of molting. The name of the pathway, its hierarchy, and the number of members plus their gene IDs are provided.
(XLSX)

S5 Table Thirty-six immunity families, including 236 immune genes, categorized between molting stages. Gene ID, number of members and expression for the eight molting stages are given for each unigene.
(XLSX)
