# Supplementary figures and images for: Whole Transcriptome Analysis Provides Insights into Molecular Mechanisms for Molting in Litopenaeus vannamei
Source: PLoS One. 2015 Dec 9;10(12):e0144350. doi: 10.1371/journal.pone.0144350 (PMC4674093; doi:10.1371/journal.pone.0144350)

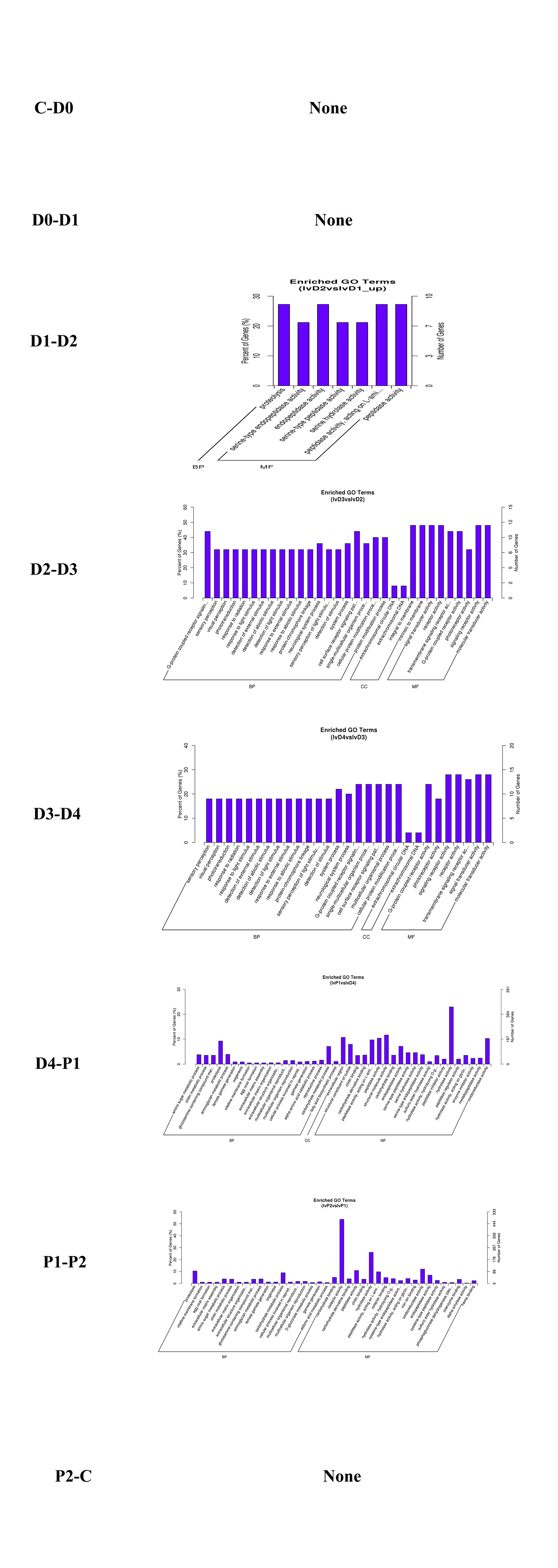

Supplement: S2 Fig — The results are summarized in three main GO categories. The x-axis represents the names of these GO subcategories. The left y-axis indicates the percentage of genes. The right y-axis indicates the number of DEGs expressed in a given sub-category. (JPG) [file pone.0144350.s002.jpg]
