# Supplementary material for: Whole Transcriptome Analysis Provides Insights into Molecular Mechanisms for Molting in Litopenaeus vannamei
Source: PLoS One. 2015 Dec 9;10(12):e0144350. doi: 10.1371/journal.pone.0144350 (PMC4674093; doi:10.1371/journal.pone.0144350)
Supplement: S1 Table — Gene descriptions, along with product lengths in brackets, are provided. (DOCX) [file pone.0144350.s003.docx]

**S1 Table** Oligonucleotide primers of six genes for the verification experiment. Gene descriptions, along with product lengths in brackets, are provided.

| Gene ID and Description | Primer sequence |
| --- | --- |
| C78849_g1（chitinase, 245bp） | **Sense:** CATCACCGAACCTCCACC |
|  | **Anti-sense:** AAGAAGCCCAATCGCAGT |
| C78622_g1（hemocyanin, 146bp） | **Sense:** ATCGGTTAGGAATACCAGTG |
|  | **Anti-sense:** CATTGTCCGTAAGTCGTCA |
| C75219_g1（anti-lipopolysaccharide factor isoform 6, 200bp） | **Sense:** TATCTGCGTGTCGTTCTTCTT |
|  | **Anti-sense:** GCAACTGTATTTCAGGGGTC |
| C65344_g1（cuticle protein, 89bp） | **Sense:** GGCGTCGTTCTGTCCAAC |
|  | **Anti-sense:** ATTCCTGAGGCTCCCACC |
| C71202_g1（beta-1,3-glucan binding protein, 160bp） | **Sense:** GCTCCCGAACTGGCTCTA |
|  | **Anti-sense:** TTACGAGGCAACATCCAAATA |
| C68968_g1（chitinase 4 precursor, 206bp） | **Sense:** ACTCACCTCATCTTCGGCTT |
|  | **Anti-sense:** GAATAATCCTCGGAACCCTC |
